# Stearoyl-CoA Desaturase 1 Is a Key Determinant of Membrane Lipid Composition in 3T3-L1 Adipocytes

**DOI:** 10.1371/journal.pone.0162047

**Published:** 2016-09-15

**Authors:** Sergio Rodriguez-Cuenca, Lauren Whyte, Rachel Hagen, Antonio Vidal-Puig, Maria Fuller

**Affiliations:** 1 Metabolic Research Laboratories, Wellcome Trust-MRC Institute of Metabolic Science, Addenbrooke's Hospital, University of Cambridge, Cambridge, United Kingdom; 2 Genetics and Molecular Pathology, SA Pathology, Adelaide, Australia; 3 Wellcome Trust Sanger Institute, Hinxton, United Kingdom; 4 University of Adelaide, North Terrace, Adelaide, SA 5000, Australia; INRA, FRANCE

## Abstract

Stearoyl-CoA desaturase 1 (SCD1) is a lipogenic enzyme important for the regulation of membrane lipid homeostasis; dysregulation likely contributes to obesity associated metabolic disturbances. SCD1 catalyses the Δ9 desaturation of 12-19 carbon saturated fatty acids to monounsaturated fatty acids. To understand its influence in cellular lipid composition we investigated the effect of genetic ablation of SCD1 in 3T3-L1 adipocytes on membrane microdomain lipid composition at the species-specific level. Using liquid chromatography/electrospray ionisation-tandem mass spectrometry, we quantified 70 species of ceramide, mono-, di- and trihexosylceramide, phosphatidylcholine, phosphatidylethanolamine, phosphatidylglycerol, bis(monoacylglycero)phosphate, phosphatidylinositol and cholesterol in 3T3-L1 adipocytes in which a 90% reduction in *scd1* mRNA expression was achieved with siRNA. Cholesterol content was unchanged although decreases in other lipids resulted in cholesterol accounting for a higher proportion of lipid in the membranes. This was associated with decreased membrane lateral diffusion. An increased ratio of 24:0 to 24:1 in ceramide, mono- and dihexosylceramide, and sphingomyelin likely also contributed to this decrease in lateral diffusion. Of particular interest, we observed a decrease in phospholipids containing arachidonic acid. Given the high degree of structural flexibility of this acyl chain this will influence membrane lateral diffusion, and is likely responsible for the transcriptional activation of Lands’ cycle enzymes lpcat3 and mboat7. Of relevance these profound changes in the lipidome were not accompanied by dramatic changes in gene expression in mature differentiated adipocytes, suggesting that adaptive homeostatic mechanisms to ensure partial maintenance of the biophysical properties of membranes likely occur at a post-transcriptional level.

## Introduction

Stearoyl-CoA desaturase 1 (SCD1) is a Δ9 desaturase highly expressed in lipogenic tissues, such as liver and adipose tissue, which catalyses the insertion of a *cis* double bond in 12-19 carbon saturated fatty acids, thereby converting them to monounsaturated fatty acids [1,2]. Through this activity, SCD1 helps to regulate the ratio of saturated to monounsaturated acyl chains in membrane lipids influencing membrane fluidity and functionality, both essential for maintaining cellular integrity. Despite substantial efforts to establish a relationship between SCD1 and onset of the metabolic syndrome, there is limited information regarding how SCD1 may specifically affect the lipidome and metabolism of adipose tissue. To this end, we have recently described an adaptive lipid homeostatic loop in white adipose tissue mediated by Insig1 and SREBP1 regulating SCD1. This loop preserves the degree of lipid unsaturation in obese and insulin resistance human adipose tissues suggesting that maintenance of an appropriate lipid unsaturation set point is actively preserved even under conditions of severe metabolic stress [3].

Animal models have revealed disparate results about the physiological consequences of SCD1 ablation. For instance, global deletion of SCD1 reportedly protects mice from diet and leptin deficiency-induced obesity and hepatic steatosis, improves insulin sensitivity and ameliorates inflammation [4,5]. However, some of these beneficial outcomes seem to be an artefact of the defects in subcutaneous lipid homeostasis and skin integrity, ultimately resulting in excessive heat loss [6]. In fact, a white adipose tissue specific SCD1 KO mouse model did not recapitulate the lean healthy phenotype of the global KO, exhibiting increased TNFα and decreased adiponectin levels [7]. Globally, these findings reveal the complexity of crosstalk amongst peripheral organs contributing to the phenotype elicited in the SCD1 KO mouse.

Therefore, we reasoned that elucidation of the specific lipid changes induced by defective SCD1 on adipose tissue may be better understood using a simpler cellular system—the adipocyte 3T3-L1 cell line. This obviates the confounding effects of diet and/or the crosstalk between liver and adipose tissue observed in animal models and human studies on SCD activity ratios. Previous studies *in vitro* have proved that pharmacological inhibition of SCD1 during 3T3-L1 adipocyte differentiation decreases the abundance of monounsaturated fatty acids 16:1n-7 and 18:1n-7, with a concomitant increase in the abundance of saturated 16:0 and 18:0, as well as the monounsaturated 18:1n-9 [8,9]. Knockdown of human SCD, the single functional orthologue of the four mouse isoforms (SCD1-4) [10], with siRNA in human adipocytes (SCD KD) has been reported to reduce membrane fluidity, insulin sensitivity and *de novo* lipogenesis [11]. Reduced membrane fluidity was accompanied by a cognate increase in the ratio of saturated to monounsaturated acyl chains in phospholipids; with increases in 16:0 and 18:0, and corresponding decreases in 16:1 and 18:1. Nevertheless, these studies were limited since they did not reveal the type of phospholipids or address the possibility of specific lipid species preference for the limited amount of unsaturated lipids generated from residual SCD activity.

Thus, we hypothesised that SCD1 KD may differentially affect the saturation index of individual species of sphingolipids and phospholipids. To investigate this, we used SCD1 KD 3T3-L1 adipocytes and extended the findings of previous studies in two main ways. We measured individual species of ceramide, mono-, di- and trihexosylceramide, phosphatidylcholine, phosphatidylethanolamine, phosphatidylglycerol, bis(monoacylglycero)phosphate, phosphatidylinositol and cholesterol, which represents to the best of our knowledge, the most complete lipidome analysis performed upon genetic SCD1 KD manipulation of adipocytes. Our lipidomic profiling was targeted to specific membrane microdomains—isolated as detergent resistant (DRM) and detergent soluble domains—which is functionally important as different membrane microdomains have different lipid compositions and fluidity, as well as execute specialised functions [12,13].

We learned that SCD1 KD modifies the acyl chain composition of lipids, as well as the lipid composition *per se* of membrane microdomains in 3T3-L1 adipocytes. The effects of SCD1 KD are not limited to alterations in 16:0 and 18:0 fatty acids, which is suggestive of additional phospholipid and sphingolipid remodelling processes. However, these homeostatic adaptations do not appear to be driven by major changes in gene expression in mature differentiated adipocytes.

## Materials and Methods

### Materials

RNAi-Ready pSIREN-RetroQ vectors were purchased from Clontech (Mountain View, US). BOSC and 3T3-L1 cells were obtained from ATCC (Manassas, VA, US). FuGene6 transfection reagent was from Roche (Lewes, UK). Cell culture consumables, insulin, 3-isobutyl-1-methylxanthine, 1-pyrenedecanoic acid dexamethasone, puromycin, bovine calf serum, rabbit polyclonal antibody against flotillin 1 and primers were purchased from Sigma (St. Louis, MO, US). Fetal bovine serum and SuperSignal West Femto Trial Kits were purchased from Thermo Fisher Scientific (Waltham, MA, US). M-MLV Reverse Transcriptase was from Promega (Madison, WI, US). TaqMan7900 sequence detection system was purchased from Applied Biosystems (Waltham, MA, US). Western blotting gels and reagents were from Invitrogen (Carlsbad, CA, US). PVDF membranes were from Perkin Elmer (Waltham, MA, US). Horseradish peroxidise-conjugated sheep anti-rabbit antibody was from Chemicon (Victoria, Australia). Internal standards BMP 14:0/14:0 {bis(monomyristoylglycero)phosphate (S,R isomer) (ammonium salt)}, ceramide (CER) 18:1/17:0 {N-heptadecanoyl-ᴅ-*erythro*-sphingosine}, phosphatidylethanolamine (PE) 17:0/17:0 {1,2-diheptadecanoyl-*sn*-glycero-3-phosphoethanolamine}, PG 14:0/14:0 {1,2-dimyristoyl-*sn*-glycero-3-phospho-(1’-rac-glycerol) (sodium salt)} and phosphatidylserine (PS) 17:0/17:0 {1,2-diheptadecanoyl-*sn*-glycero-3-phospho-L-serine (sodium salt)} were purchased from Avanti Polar Lipids (Alabaster, AL, USA); dihexosylceramide (DHC) 18:1/16:0 (*d*_*3*_) {N-palmitoyl-*d*_*3*_-lactosylceramide}, monohexosylceramide (MHC) 18:1/16:0 (d_*3*_) {N-palmitoyl-*d*_*3*_-glucocerebroside}, phosphatidylinositol (PI) 16:0/16:0 {phosphatidylinositol, dipalmitoyl (NH_4_^+^ salt)} and trihexosylceramide (THC) 18:1/17:0 {N-heptadecanoyl ceramide trihexoside} were from Matreya LLC (Pleasant Gap, PA, USA); cholesterol ester (CE) 17:0 {cholesterol heptadecanoate} and phosphatidylcholine (PC) 14:0/14:0 {1,2-dimyristoyl-*sn*-glycero-3-phosphocholine} were from Sigma (St. Louis, MO, USA). All solvents were of HPLC grade except chloroform, which contained 1% ethanol and was reagent grade, and were used without further purification.

### Generation of the SCD1 KD cell line

RNAi-Ready pSIREN-RetroQ vectors were used to target SCD1 in 3T3-L1 cells. Oligonucleotides containing short sequences targeting SCD1 were ligated into the pSIREN vector following the manufacturer’s protocols. A control vector was also generated using the sequence GTGCGTTGCTAGTACCAAC, which is not specific for any known mouse mRNAs. Vectors were then sequenced to ensure insertion of the correct sequence. Retroviruses were generated by transiently transfecting BOSC cells with the pSIREN plasmids using FuGene6 transfection reagent. FuGene6 is a multi-component reagent composed of lipids forming a complex with the DNA to be inserted and transports it into the desired cells. Five μg of DNA was complexed with 23 μl of Fugene 6 reagent in 500 μl of OPTIMEM and incubated at room temperature for 20 minutes. The Fugene 6:DNA complex was then added to the media of the flask containing BOSC cells. Forty-eight hours post-transfection, the viral supernatant was filtered through a 0.45 μm filter, added to fresh media and polybrene (4 μg/ml) and used to infect 3T3-L1 cells that had been seeded at 40% confluence the day before in 25 cm^2^ flasks. Twenty-four hours post-retroviral infection cells carrying the plasmid were selected with puromycin (4 μg/ml). After a preliminary experiment confirming the knockdown of SCD1 mRNA, the stable cell lines were seeded into flasks to generate enough inoculum to freeze and perform experiments as detailed below.

### Cell culture

Control 3T3-L1 carrying the scramble sequence and SCD1 knockdown (SCD1 KD) pre-adipocytes were maintained in growth medium (high glucose DMEM supplemented with 10% bovine calf serum, 100 U/ml penicillin and 100 μg/ml streptomycin). During proliferation, pre-adipocytes were sub-cultured using trypsin and not allowed to reach confluence. For differentiation into adipocytes, pre-adipocytes were grown to two days post confluence and growth medium was replaced with differentiating medium (growth medium with 10% fetal bovine serum instead of bovine calf serum) containing 10 μg/ml insulin, 0.5 mM 3-isobutyl-1-methylxanthine and 1 μM dexamethasone (day 0). Medium was replenished every three days thereafter with differentiating medium containing 10 μg/ml insulin. For SCD1 KD pre-adipocytes and adipocytes, puromycin (4 μg/ml) was added to all culture media.

### Visualisation of cellular lipid content *via* Oil Red O staining

On day 8, following induction of differentiation, mature differentiated adipocytes were fixed with 0.5% glutaraldehyde for 5 min, washed briefly in 60% isopropanol and stained with Oil Red O for (ORO) 30 min. Excess stain was removed and cells briefly washed with 60% isopropanol and then stored in PBS at 4°C.

### Measurement of short range lateral diffusion

Lateral diffusion of the acyl chains was measured by the intermolecular excimerization of the fluorescent probe pyrene and based on the protocol in Masuda et al. [14]. Briefly, 3T3-L1 pre-adipocytes were grown to confluence, gently scraped from a 75 cm^2^ flask and 5,000 pre-adipocytes were incubated in the presence of 20 μM 1-pyrenedecanoic acid at 37°C for 1 h. Excess probe was removed and the cells washed twice with PBS. Cells were resuspended in 1 ml PBS and 200 μl aliquoted into a 96-well plate. Fluorescent measurement was captured using an excitation wavelength of 345 nm, and emission wavelength of 372 nm (monomer) and 470 nm (excimer). The ratio of monomer to excimer was then calculated, with a higher ratio reflecting greater microviscosity.

### RNA isolation and Real-Time PCR

RNA was extracted from cells at the time points indicated using the Qiagen RNeasy kit in accordance with manufacturer's instructions. One microgram of RNA was converted to cDNA using M-MLV Reverse Transcriptase, 100 ng random hexamers and 1 mmol/l dNTPs. Real-Time PCR was performed using SybrGreen primers (300 nmol) and reactions were carried out using ABI 2X SybrGreen Master Mix in a TaqMan7900 Sequence detection system. Primers were designed using Primer Express 2.0 software and sequences are available at http://tvp.mrl.ims.cam.ac.uk/primer-database-pagemax. The geometrical average of four different genes (β2 microglobulin, β-actin, 18S and 36B4) was used as an internal control for data normalisation.

### Isolation of membrane microdomains

Control adipocytes (containing the scramble sequence) and SCD1 KD adipocytes were harvested from one 100 x 20 mm culture dish at day 10 after induction of differentiation. Cell pellets were used directly for isolation of membrane microdomains, as detergent resistant membranes (DRM) and detergent soluble membranes using the method of Lisanti et al. [15]. Briefly, cell pellets were resuspended in 2 ml of MES-buffered saline (MBS; 25 mM MES pH 6.5, 0.15 M NaCl) containing 1% (v/v) Triton X-100 and 1 mM PMSF, and homogenised with 12 strokes of a Dounce homogeniser. Samples were incubated on ice for 30 min and then centrifuged at 425 *g* for 5 min at 4°C to remove cellular debris. Total cell protein was determined using a 50 μl aliquot of the supernatant using the method of Lowry et al. [16]. The supernatant was placed in a Beckman (Palo Alto, CA, US) centrifuge tube (14 mm x 95 mm) and 2 ml of 80% (w/v) sucrose in MBS containing 1% (v/v) Triton X-100 and 1 mM PMSF, was used to adjust the sucrose concentration to 40% (w/v). A sucrose gradient was formed by gently layering 5 ml of 30% (w/v) sucrose in MBS containing 1 mM PMSF onto the sample, followed by 3 ml of 5% (w/v) sucrose in MBS containing 1 mM PMSF. Samples were centrifuged at 270,519 *g* for 16-20 h at 4°C using a swing-out rotor and 12 x 1 ml fractions were collected from the top of the gradient. DRM were characterised by Flotillin-1 and cholesterol as described previously [17].

### Lipid isolation and quantification

Lipids were extracted from 750 μl of each membrane fraction using the method of Bligh and Dyer [18], with the inclusion of 400 pmol of the following internal standards: BMP 14:0/14:0, CER 18:1/17:0, CE 17:0, DHC 18:1/16:0(*d*_*3*_), MHC 18:1/16:0(*d*_*3*_), PC 14:0/14:0, PE 17:0/17:0, PG 14:0/14:0, PI 16:0/16:0, PS 17:0/17:0 and THC 18:1/17:0. Dried lipid extracts were resuspended in 0.2 ml CH_3_OH containing 10 mM NH_4_COOH and individual species of sphingolipids and phospholipids were quantified by LC/ESI-MS/MS using multiple reaction monitoring on a PE SCIEX API 4000 Q-trap mass spectrometer as previously described [19], with the inclusion of the 18:0/20:0, 18:1/16:0, 18:1/16:1, 18:1/18:1, 18:1/24:0 and 18:1/24:1 species of SM, the 36:4 and 38:4 species of PC and the 16:1/16:0 species of BMP. SM used the *m/z* product ion of 184 corresponding to the phosphocholine head group and the peak areas were related to the peak area of the PC 14:0/14:0 internal standard. For HPLC separation of SM and PC, a longer gradient than previously described was used to facilitate better peak resolution. For SM and PC, the Alltima C18 column was equilibrated in 75% mobile phase A (30% tetrahydrofuran/20% CH_3_OH/50% H_2_O in 5 mM NH_4_COOH) and then linearly converted to 100% mobile phase B (70% tetrahydrofuran/20% CH_3_OH/10% H_2_O in 10 mM NH_4_COOH) over 15 min and maintained there for 2 min. The column was re-equilibrated with 75% mobile phase A for 3 min prior to the next injection.

Cholesterol was determined in each of the membrane microdomain fractions following lipid extraction (as above) by converting the cholesterol in each sample to cholesteryl ester with the addition of 100 μl acetyl chloride-CHCl_3_ 1:5 (v/v) and analyzed by LC/ESI-MS/MS [20]. Concentrations of cholesterol were determined by relating the peak area of cholesterol to the peak area of the internal standard.

### Statistics

All data are presented as mean ± standard deviation. For gene expression data, a one-way ANOVA was used to determine statistical significance with p < 0.05 considered significant. For all other data, the Student’s *t*-test was used to determine statistical significance with p < 0.05 considered significant.

## Results

### SCD1 KD preadipocytes undergo normal adipogenesis and lipid accumulation

**Fig 1:**
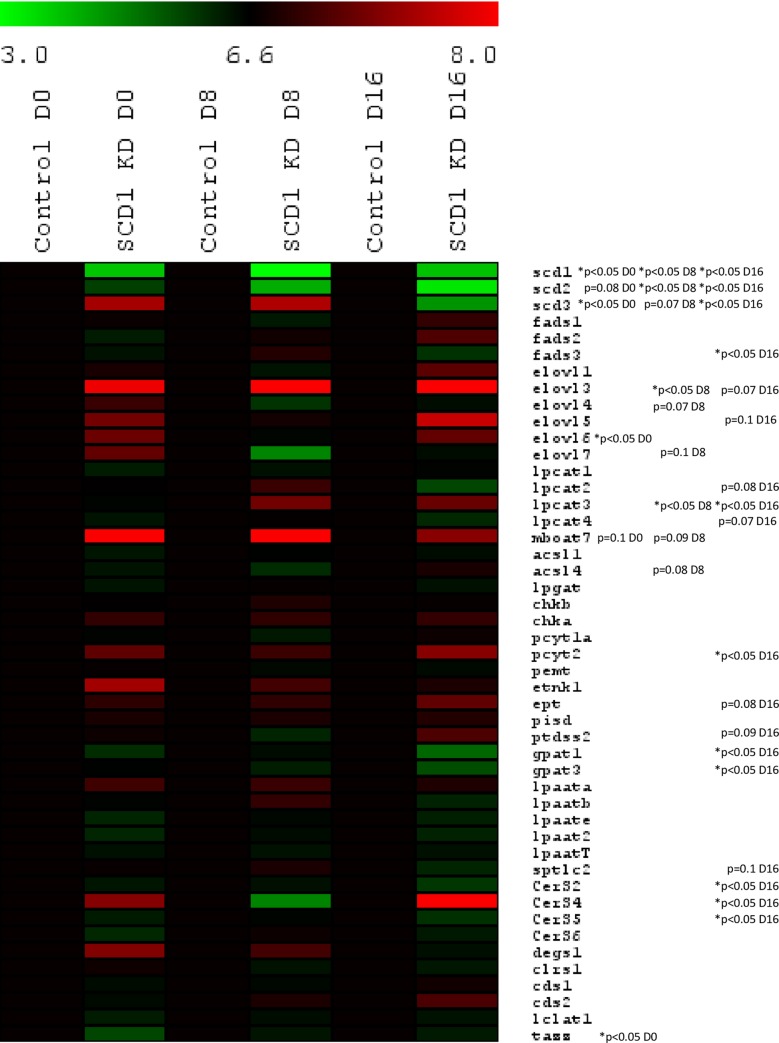
Real-Time PCR gene expression during differentiation of control and SCD1 KD adipocytes. Gene expression (n = 6 from 2 independent experiments) is shown as log2 conversions of average gene expression data relative to control (log2 100 = 6.6). Magnitude > 6.6 and < 6.6 denotes up- and downregulation, respectively, compared with controls. Expression is reported for day 0 (D0), day 8 (D8) and day 16 (D16) after induction of differentiation. *Significance determined by one-way ANOVA.

**Fig 2:**
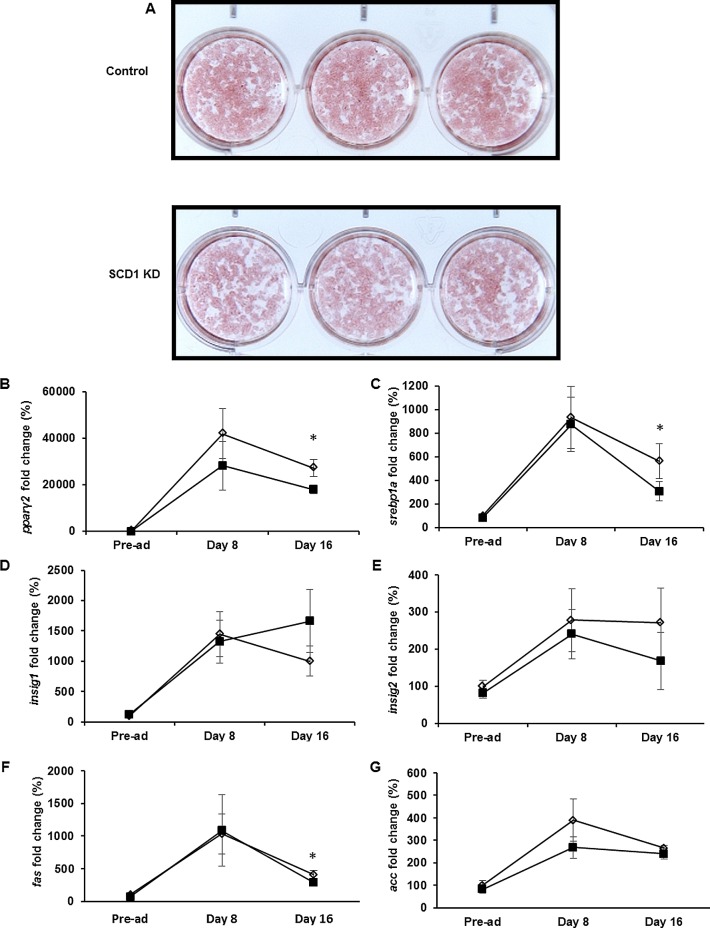
Adipogenesis in control and SCD1 KD adipocytes. Oil Red O staining was performed at day 8 after induction of differentiation (A). RNA expression profile of lipogenic genes during differentiation at day 0 (Pre-ad), day 8 and day 16 is shown in B. Results are expressed as the mean and standard deviation (n = 6 from two independent experiments) for control (open diamonds) and SCD1 KD (filled squares) adipocytes. *Significant at p<0.05 (one-way ANOVA).

**Fig 3:**
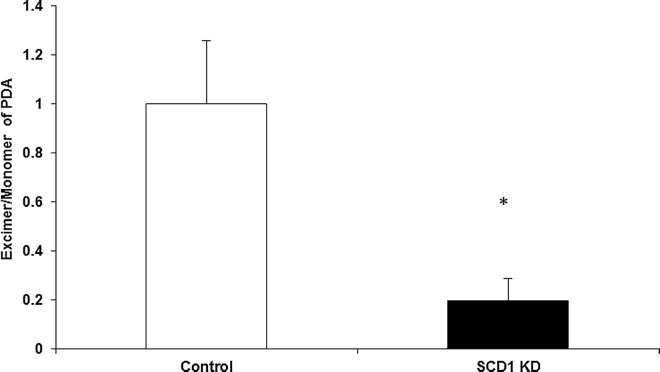
Measurement of short range lateral diffusion using 1-pyrenedecanoic acid (PDA). Ratio of excimer and monomer of PDA for control (open bars) and SCD1 KD (filled bars) pre-adipocytes. Results are expressed as the mean and standard deviation (n = 6 from two independent experiments). *Significant at p<0.05 (Student's *t*-test).

SiRNA targeting *scd1* caused a 90% reduction in the expression of *scd1* mRNA (Fig 1). Interestingly, knockdown of *scd1* did not significantly impair lipogenesis/fat deposition during 3T3-L1 adipogenesis, as observed by Oil Red O staining of mature differentiated adipocytes (day 8 after induction of differentiation) (Fig 2A). Preservation of the lipogenic capacity was consistent with no differences at the gene expression level of lipogenic genes measured at this time (Fig 2B–2G). Interestingly, mild decreases in *pparγ*2, *srebp*1a and *fas* were only observed in post-mature differentiated adipocytes (day 16 after induction of differentiation) (Fig 2B, 2C and 2F), likely reflecting a time point when the homeostatic responses activated in SCD1 KD, likely directed to preserve “normal lipid composition”, were compromised. Despite the apparent normal lipid deposition in mature differentiated adipocytes, SCD1 KD pre-adipocytes were not normal as they exhibited defective membrane lateral diffusion (Fig 3) indicating that SCD1 activity and maintenance of unsaturated fatty acid levels are important for membrane integrity [21].

### Genetic ablation of SCD1 increased the expression of SCD3

Given the normal differentiation we evaluated the existence of compensation to impaired expression of SCD1 by other SCD family members. An increase in *scd*3 expression in pre-adipocytes (day 0) and mature differentiated adipocytes (day 8), followed by down-regulation later on in post-mature differentiated adipocytes (day 16) was observed (Fig 1). *Scd*2 expression was down-regulated during the entire differentiation process (Fig 1).

### Membrane microdomain lipid and acyl chain composition are altered in SCD1 KD

**Fig 4:**
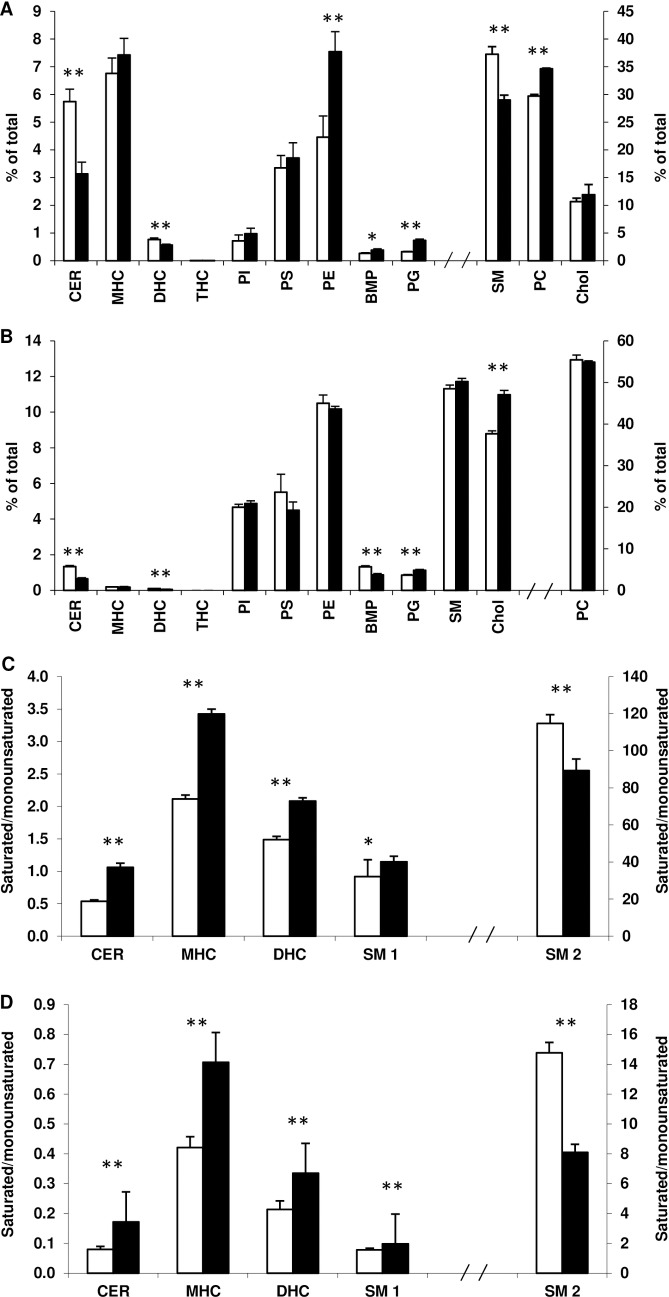
Lipid composition of membrane microdomains in control and SCD1 KD adipocytes. Individual lipid species in fractions 3 and 4 (DRM) and in fractions 7-12 (soluble) domains were summed. All species in the DRM (A) or soluble domains (B) were then summed for each lipid and expressed as a percentage of the total. The percentage of CER, MHC, DHC, THC, SM, PC, PI, PS, PE, BMP, PG and cholesterol (Chol) is shown. The ratios of 18:1/24:0 to 18:1/24:1 for CER, MHC, DHC and SM (SM 1) and 18:1/16:0 to 18:1/16:1 for SM (SM2) are shown in the DRM (C) and soluble membrane domains (D). Results for control (open bars) and SCD1 KD (filled bars) adipocytes are expressed as the mean and standard deviation (n = 3). *Significant at p<0.05, **significant at p<0.01 (Student’s *t*-test).

To investigate the effect of genetic ablation of SCD1 on membrane lipid composition, membrane microdomains were isolated as DRM and localised to fractions 3 and 4 by the presence of flotillin 1 and cholesterol, distinct from the soluble domains in fractions 7-12 (S1 Fig). SCD1 KD adipocytes showed the same flotillin 1 distribution as the control adipocytes (data not shown). Fig 4A shows that SM, PC and cholesterol were the predominant lipids in the DRM, collectively comprising more than 75% of DRM in both control and SCD1 KD adipocytes. Fig 4A also shows that in absolute terms, cholesterol levels were similar in the control and SCD1 KD adipocytes. However, the percentage of CER, DHC and SM decreased in the DRM of SCD1 KD adipocytes compared to control adipocytes, whereas the percentage of PC, PE, BMP and PG increased. PC was the predominant lipid in the soluble domains of both control and SCD1 KD adipocytes, comprising more than 55% of the lipids measured in these domains (Fig 4B). Unlike the DRM, there was an increase in the percentage of cholesterol in the soluble domains of the SCD1 KD adipocytes compared to control (Fig 4B). The percentage of the soluble domains comprised of PG also increased in the SCD1 KD adipocytes, whereas the percentage of CER, DHC and BMP decreased (Fig 4B). Importantly, the ratio of PC to PE in the soluble domains was maintained, as alterations in this ratio have been associated with membrane leakage in other models [22].

**Fig 5:**
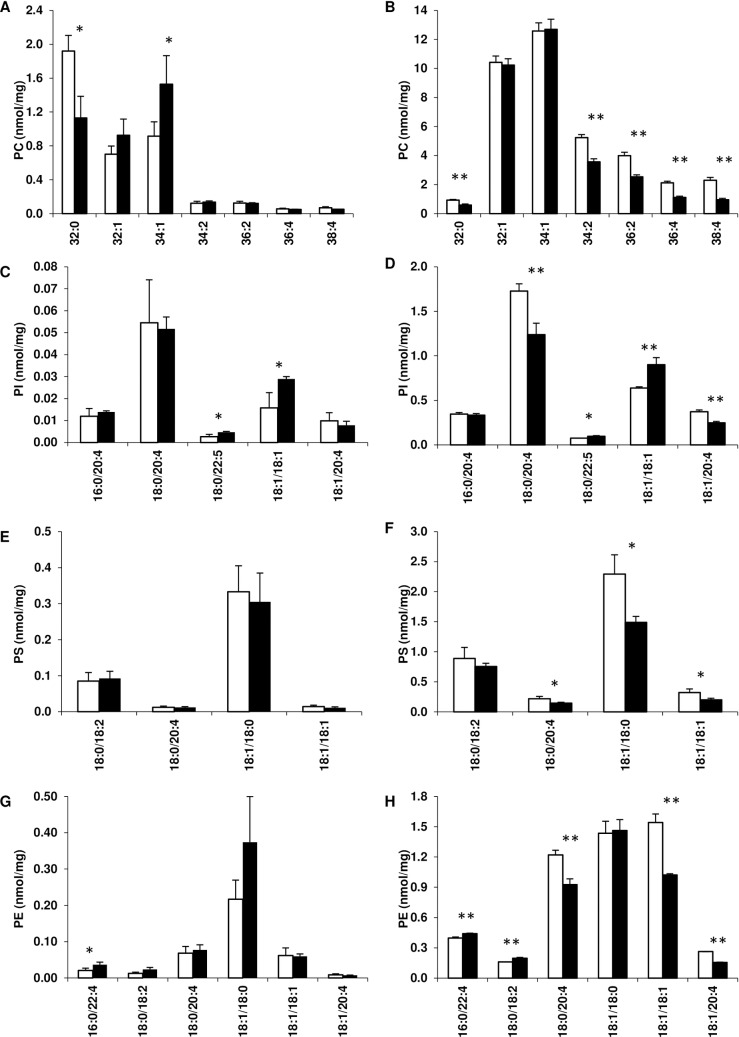
Phospholipids in the membrane microdomains of control and SCD1 KD adipocytes. The concentration of individual species of PC, PI, PS and PE are shown for DRM (A, C, E, G) and soluble domains (B, D, F, H). Results for control (open bars) and SCD1 KD (filled bars) adipocytes are expressed as the mean and standard deviation (n = 3) in nmol per mg of protein loaded into the sucrose gradient prior to fractionation.*Significant at p<0.05, **significant at p<0.01 (Student’s *t*-test).

We next investigated whether the acyl chains in sphingolipids and phospholipids present in the membrane microdomains were altered in SCD1 KD. Firstly we considered the ratios of saturated acyl chains to structurally similar monounsaturated acyl chains of CER, MHC, DHC and SM. For all of these lipids the ratio of 18:1/24:0 to 18:1/24:1 increased in the DRM (Fig 4C) and soluble domains (Fig 4D) of SCD1 KD adipocytes compared to controls. Conversely, the ratio of SM 18:1/16:0 to 18:1/16:1 decreased in the DRM (Fig 4C) and soluble domains (Fig 4D) of SCD1 KD adipocytes as expected from KD of SCD1. Secondly, the acyl chains in the phospholipids PC, PI, PS and PE were examined and predominately (> 77%) found in the soluble domains for both control and SCD1 KD adipocytes, with the exception of the fully saturated species, PC 32:0, which predominated in the DRM (S2 Fig). PC 32:0 decreased in both the DRM and soluble domains of SCD1 KD adipocytes (Fig 5A and 5B). The only other species of PC measured—differing in the DRM of SCD1 KD adipocytes—was 34:1, which was increased in the SCD1 KD adipocytes compared to control (Fig 5A). In the soluble domains, PC 34:2, 36:2, 36:4 and 38:4 were all decreased in SCD1 KD adipocytes compared to control, while PC 32:1 and 34:1 were unchanged (Fig 5B). For PI, PS, and PE, all species containing arachidonic acid (AA, 20:4) decreased in the soluble domains of SCD1 KD adipocytes compared to control, with the only exception of PI 16:0/20:4, which was preserved (Fig 5D, 5F and 5H). Species containing AA were present in much lower amounts in the DRM, and were not significantly different between control and SCD1 KD adipocytes (Fig 5C, 5E and 5G). The remaining species of PI measured (18:0/22:5 and 18:1/18:1) were increased in the DRM and soluble domains of SCD1 KD adipocytes compared to control (Fig 5C and 5D). Conversely, the 18:1/18:1 species of PS and PE were decreased in the soluble domains of SCD1 KD adipocytes (Fig 5F and 5H). These species were present in much lower amounts in the DRM, and were not significantly different between control and SCD1 KD (Fig 5E and 5G). PS 18:1/18:0 was also decreased in the soluble domains of SCD1 KD adipocytes, but unchanged in the DRM (Fig 5E and 5F).

### SCD1 KD alters the amount and acyl chain composition of the structural isomers, PG and BMP

**Fig 6:**
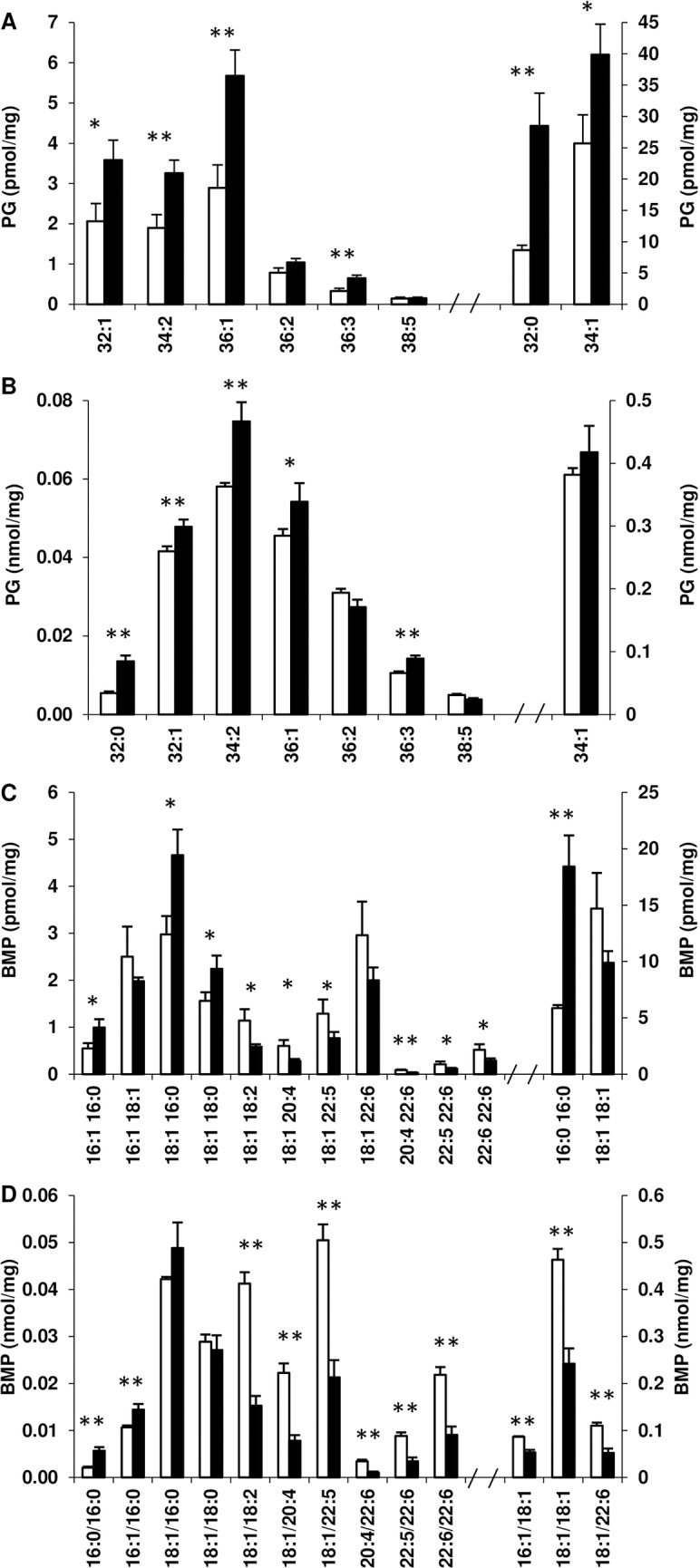
PG and BMP in the membrane microdomains of control and SCD1 KD adipocytes. The concentration of individual species of PG (A, B) and BMP (C, D) in the DRM (A, C) and soluble membrane domains (B, D) for control (open bars) and SCD1 KD (filled bars) adipocytes are shown. Results are expressed as the mean and standard deviation (n = 3) in nmol or pmol per mg of protein loaded into the sucrose gradient prior to fractionation. *Significant at p<0.05, **significant at p<0.01 (Student’s *t-*test).

Like the phospholipids discussed above, PG and BMP were found predominately (> 90%) in the soluble domains in both, control and SCD1 KD adipocytes Again the exceptions were the fully saturated species, PG 32:0 and BMP 16:0/16:0, predominating in the DRM (S3 Fig). These saturated species increased in both microdomains of SCD1 KD adipocytes compared to control, both in total amount (Fig 6), and relative to their monounsaturated counterparts: PG 32:1 and BMP 16:0/16:1 (S4 Fig). All species of PG measured increased in the DRM and soluble domains of SCD1 KD adipocytes compared to control, except for 36:2 and 38:5, which were not significantly different (Fig 6A and 6B), and 34:1 which was increased in the DRM (Fig 6A), but not in the soluble domains (Fig 6B).

**Fig 7:**
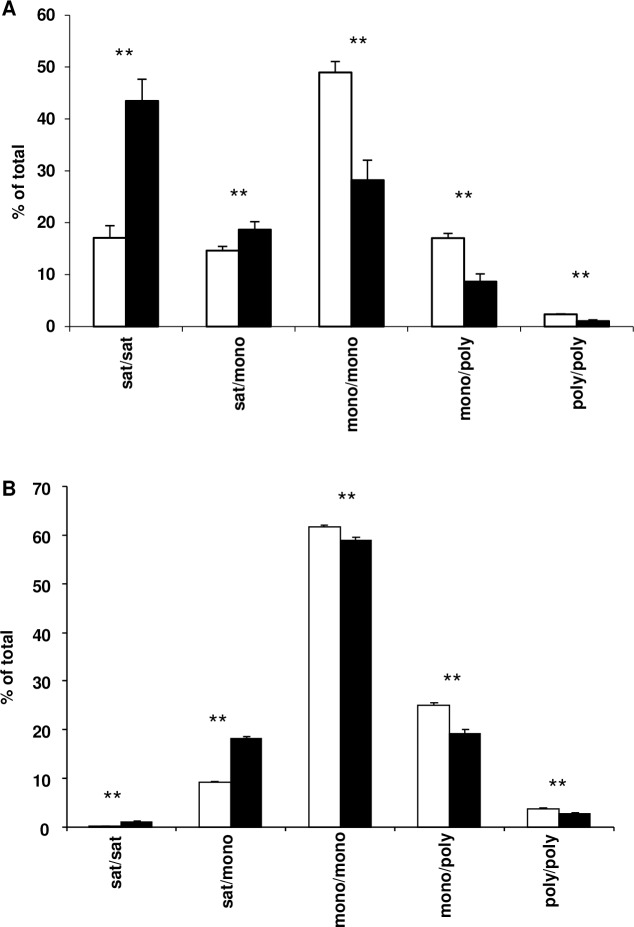
Fatty acid composition of BMP in the membrane microdomains of control and SCD1 KD adipocytes. Species of BMP with saturated/monounsaturated, mono-/monounsaturated, mono-/polyunsaturated and poly-/polyunsaturated fatty acids in the DRM (A) and soluble membrane domains (B) were summed and along with the fully saturated species expressed as a percentage of the total BMP in the DRM or soluble membrane domains. Results for control (open bars) and SCD1 KD (filled bars) adipocytes are expressed as the mean and standard deviation (n = 3).

The total amount of BMP was not significantly different in the DRM of SCD1 KD adipocytes (0.035 nmol/mg in control compared to 0.042 nmol/mg in SCD1 KD), but the amount of BMP in the soluble domains decreased from 0.9 nmol/mg in control adipocytes to 0.5 nmol/mg in SCD1 KD adipocytes. The acyl chain composition of BMP was altered in both DRM and soluble domains. All species of BMP measured containing a saturated acyl chain increased in the DRM of SCD1 KD adipocytes compared to control, while all species containing a polyunsaturated acyl chain decreased, except for 18:1/22.6, which was not significantly different (Fig 6C). The reduction of BMP in the soluble domains was caused by a decrease in all species measured with both acyl chains unsaturated (Fig 6D). The 16:0/16:0 and 16:1/16:0 species were the only species of BMP to increase significantly in the soluble domains of the SCD1 KD adipocytes (Fig 6D). Fig 7 shows that the percentage of BMP containing fully saturated and saturated/monounsaturated acyl chains increased in the membrane microdomains of the SCD1 KD adipocytes, whereas the percentage with mono-/monounsaturated, mono-/polyunsaturated and poly-/polyunsaturated acyl chains decreased.

### SCD1 KD altered the expression of genes involved in long chain fatty acid (LCFA) biosynthesis

Given that the lipid composition of the membrane domains was altered in SCD1 KD adipocytes, we investigated the expression of genes involved in lipid remodelling. First, we considered the possibility that biosynthesis of LCFA could have been compromised based on our finding that AA-enriched phospholipids were decreased in SCD1 KD adipocytes (Fig 5). Interestingly, *elov*l6 was upregulated in SCD1 KD pre-adipocytes (day 0) followed by a sustained increase in *elov*l3 throughout differentiation (Fig 1). Expression of *elovl*4 and 7 tended to be down-regulated in mature differentiated adipocytes (day 8) (Fig 1). Together, these data suggest that activation of specific elongation pathways may be considered as an adaptive mechanism aimed to diversify the amount of saturated 16:0 and 18:0 to other, specific, longer, and likely less harmful, fatty acid species. *Fads*1 and *fads*2, the major desaturases involved in the biosynthesis of long chain polyunsaturated fatty acids were not modulated at the gene expression level (Fig 1). Interestingly, the expression of *fads*3, a Δ13 desaturase [23] was downregulated in post-mature differentiated adipocytes (day16) suggesting dysfunctional SCD1 may indirectly impair unsaturation of additional complex lipids.

### SCD1 KD activates the expression of enzymes involved in the remodelling of phospholipids (Lands cycle)

We investigated whether Lands cycle phospholipid remodelling pathways were deregulated in SCD1 KD adipocytes. Fig 1 depicts an increase in the expression of both *lpcat*3 and *mboat*7 (p = 0.09) in SCD1 KD mature differentiated adipocytes (day 8). *lpcat*3 is mostly responsible for the re-acylation of linoleic acid (18:2) and AA (20:4) into lysophosphatidylcholine, whereas *mboat*7 shows preference for the re-acylation of AA into lysophosphatidylinositol. These alterations are consistent with changes in the AA pool and the activation of homeostatic mechanisms aimed to preserve specific subsets of AA enriched phospholipids (PC and PI).

Finally, we measured the expression of genes involved in the biosynthesis of phospholipids to evaluate the impact of *scd*1 depletion on the overall regulation of synthesis of phospholipid species at a transcriptional level. Surprisingly, no major changes were observed during differentiation of SCD1 KD adipocytes. We did note down-regulation of *gpat*1 and 3 in post-mature differentiated adipocytes (day 16) (Fig 1). This may lead to a decrease in the repertoire of lipid species by directly regulating the expression of enzymes that show preference for specific fatty acids as a substrate of their acylation reactions during the conversion of glycerol-3-phosphate to lysophosphatidic acid and phosphatidic acid, respectively. We also observed an increase in the expression of *ptdss*2, *ept* and *pcyt*2—genes involved in the synthesis of PS and PE phospholipids respectively, in post-mature differentiated adipocytes (day 16) (Fig 1)—suggesting that in late stages of differentiation the biosynthesis of the aforesaid phospholipids may be favoured. Globally, these data indicate that the defect in SCD1 expression only becomes evident at the transcriptional level for the biosynthesis of phospholipids in post-mature differentiated adipocytes, likely reflecting the collapse of additional homeostatic responses (such as enzymatic activities and control of metabolic fluxes).

### SCD1 KD increases the expression of ceramide synthase 4 at late stages of differentiation

Palmitate (16:0) is a necessary substrate for the synthesis of CER, and levels of CER are expected to increase when levels of palmitate are high. However, no major changes in expression of genes involved in the *de novo* synthesis of CER were observed during differentiation of SCD1 KD adipocytes (Fig 1). Nevertheless, we did observe an increase in the expression of *cerS*4 accompanied with decreased expression of *cerS*2 and *cerS*5 in post-mature differentiated adipocytes (Fig 1). These results suggest that the biosynthetic flux of CER is not regulated transcriptionally during normal differentiation in SCD1 KD cells, becoming only relevant in post-mature differentiated adipocytes to accommodate the likely increased levels of 18:0 as a result of the increased expression of *elov*l6, or alternatively, to favour the synthesis of specific subsets of CER and/or SM.

## Discussion

Ablation of SCD1 in adipocytes causes a substantial alteration in the acyl chain composition of phospholipids and sphingolipids, which is associated with a decrease in membrane lateral diffusion. However, the lipogenic programme was robustly preserved during adipocyte differentiation, likely at the expense of activation of homeostatic mechanisms aimed to compensate for the ablation of SCD1. The profound changes observed in the lipidome of membrane microdomains were not driven by dramatic changes in the transcriptome, suggesting that the adaptive response to modulate membrane dynamics in SCD1 KD cells is mostly undertaken at the post-transcriptional level. Only in late post-mature differentiated adipocytes (day 16) changes in the transcriptome of relevant genes in lipogenesis, as well as in phospholipid and sphingolipid metabolism, were observed, likely reflecting an attempt to limit/sustain the synthesis of specific subsets of complex lipids.

The observed decrease in lateral diffusion upon SCD1 KD (Fig 3) was most likely due to the increased proportion of cholesterol in the soluble domains (Fig 4B). The increased ratio of lignoceric (24:0) to nervonic (24:1) acid observed in CER, MHC, DHC and SM in the DRM (Fig 4C) and soluble domains (Fig 4D) of SCD1 KD adipocytes may also have contributed to the decrease in lateral diffusion. Given that lignoceric acid is not a direct known substrate of SCD1 [1], changes in 24:0/24:1 ratio may result from alteration in the elongation pathways of 18:0 and/or 18:1 [24]. The increased levels of *elov*l3 expression observed in SCD1 KD adipocytes (Fig 1) may account, at least in part for those changes.

It is also interesting to note the decrease in the global percentage of SM, CER and DHC, and the concomitant increase in the percentage of PC and PE, but also the trend for PS and PI in the DRM of SCD1 KD adipocytes (Fig 4A). This “switch” in the balance of sphingolipids and phospholipids may be important as decreased CER and SM within the DRM has been shown to affect insulin signaling, given the importance of this membrane domain for insulin signaling and the location of the insulin receptor therein [25]. Further work is required to address this.

The decreased ratio of PC 32:0 to 32:1 (Fig 5A and 5B) and SM 18:1/16:0 to 18:1/16:1 (Fig 4C and 4D) in the membrane domains of SCD1 KD adipocytes may also be a mechanism by which the cell attempts to compensate/ameliorate the decreased fluidity. Whilst we were not able to confirm the individual acyl chains present in PC 32:0 and 32:1, it is likely that they were predominantly palmitic (16:0) and palmitoleic (16:1) acid [26]. This result is somewhat surprising given that SCD KD in cultured human adipocytes has been shown to decrease the ratio of 16:1 to 16:0 in total phospholipids [11]. Nevertheless, gene expression profiling revealed an up-regulation of *scd*3 that, along with residual expression of SCD1, could explain the maintenance of certain levels of 16:1. Globally, this may be interpreted as the activation of homeostatic pathways aimed to counterbalance the changes in membrane fluidity driven by the ablation of SCD1. Our data show that the decreased ratio of 16:0 to 16:1 may be specific to SM and PC, given that PG 32:0 increased relative to PG 32:1, and BMP 16:0/16:0 increased relative to 16:1/16:0 in both DRM and soluble domains in SCD1 KD adipocytes (S4 Fig). This suggests that the synthesised monounsaturated 16:1 or 18:1 may be specifically targeted to a subset of lipids, such as SM. The decrease in PS and PE 18:1/18:1 in the soluble domains of SCD1 KD adipocytes, compared to the increase in PI 18:1/18:1 (Fig 5D, 5F and 5H), further supports our hypothesis that SCD1 KD does not result in an homogeneous decrease in the unsaturation index in all lipid species, but may divert monounsaturated fatty acids to specific subsets of phospholipids.

One of the most relevant findings of this study is the specific decrease in AA-enriched PI, PS, PE and likely PC in SCD1 KD adipocytes (Fig 5B, 5D, 5F and 5H). Whilst we were unable to determine the sn-1/sn-2 acyl chain composition of PC 36:4 and 38:4, it is likely that they contained AA [26,27]. This is not the first study to report alterations in polyunsaturated acyl chains as a result of alterations to SCD1. Interestingly, Ralston et al. found an increase in AA-enriched phospholipids upon SCD1 inhibition, but this was at day 7 of differentiation, from total cellular extracts, and with ~51% inhibition of SCD1 [9]. This may indicate that reductions in AA-enriched phospholipids only occur only upon severe reduction in SCD1. A direct relationship between SCD1 activity and AA, as reported herein, is supported by Rogowski et al., demonstrating an increase in the polyunsaturated content of triglyceride in skeletal muscle in association with SCD1 overexpression [28]. The decrease in AA enriched phospholipids was unlikely to be due to a failure in PUFA biosynthesis, as there were no increases in phospholipids containing linoleic acid. However, it is likely to be an additional contributor to reduced membrane lateral diffusion because AA has a high degree of structural flexibility. The concept of changes in the turn-over of AA is further supported by the transcriptional activation of *lpcat*3 and *mboat*7 (Fig 1) both enzymes involved in phospholipid remodelling whose role is to enrich PC and PI with AA in the sn2 moiety, respectively. We anticipate this may act as a compensatory mechanism to divert scarce AA available to preserve adequate levels of specific AA-enriched PC and PI.

The alterations in BMP (Figs 6 and 7) and PG (Fig 6), which are primarily present in the inner membranes of late endosomes and lysosomes [29,30] and in mitochondrial membranes [31], respectively, are noteworthy as they suggest the role of SCD1 is not limited to the plasma membrane, and that SCD1 may affect intracellular membranes and associated trafficking events.

In conclusion, we provide evidence of both quantitative and qualitative changes that take place in the adipocyte lipidome in response to the genetic ablation of SCD1. First, we identified a subset of phospholipids enriched in monounsaturated acyl chains, suggesting the existence of cellular mechanisms that preferentially direct the limited amount of monounsaturated fatty acids available in SCD1 KD adipocytes towards specific lipid species. We believe that this provides evidence for the existence of novel regulatory lipid homeostatic loops to preserve cellular homeostasis. Secondly, we observed a decrease in the sphingolipid/phospholipid ratio, and a specific decrease in AA-enriched phospholipids. Given the role of increased sphingolipids in the pathophysiology associated with obesity and AA as precursor to key inflammatory mediators, n-6 eicosanoids, prostaglandins and leukotrienes [32], it is conceivable that some of the beneficial effects related to the pharmacological and genetic inhibition of SCD1 reported in the context of obesity [33–35] are due to the reduction of those metabolites. This is something that warrants further investigation.

## Supporting Information

S1 Fig Distribution of flotillin 1 and cholesterol in membrane microdomains. Western blot showing flotillin 1 for each of the membrane fractions isolated from control adipocytes (A). Cholesterol is shown for control (open diamonds) and SCD1 KD (filled squares) adipocytes in B. Results are expressed as mean and standard deviation (n = 3) in nmol of cholesterol per mg of protein loaded onto the gradient prior to fractionation.
(TIF)

S2 Fig Distribution of phospholipids. Membrane microdomains were isolated from control 3T3-L1 and SCD1 KD adipocytes and individual species of phospholipids are depicted across the 12 fractions. PC 32:0 (closed diamonds), 32:1 (open squares), 34:1 (closed triangles), 34:2 (crosses), 36:2 (closed circles), 36:4 (open circles) and 38:4 (open triangles) are shown in A (control) and B (SCD1 KD). PI 16:0/20:4 (closed diamonds), 08:0/20:4 (open squares), 18:0/22:45 (closed triangles), 18:1/18:1 (crosses), 18:1/20:4 (closed circles) are shown in C (control) and D (SCD1 KD). PS 18:0/18:2 (closed diamonds), 18:0/20:4 (open squares), 18:1/18:0 (closed triangles), 18:1/18:1) are shown in E (control) and F (SCD1 KD). PE 16:0/22:4 (closed diamonds), 18:0/18:2 (open squares), 18:0/20:4 (closed triangles), 18:1/18:0 (crosses), 18:1/18:1 (open circles), 18:1/20:4 (closed squares) are shown in G (control) and H (SCD1 KD). Mean results are expressed (n = 3) in nmol or pmol of protein loaded onto the sucrose gradient prior to fractionation.
(TIF)

S3 Fig Distribution of BMP and PG. Membrane microdomains were isolated from control 3T3-L1 and SCD1 KD adipocytes and individual species of phospholipids are depicted across the 12 fractions. BMP 16:0/16:0 (closed diamonds), 16:1/16:0 (open squares), 16:1/18:1 (closed triangles), 18:1/16:0 (crosses), 18:1/18:0 (closed squares), 18:1/18:2 (asterisks), 18:1/18:1 (open circles), 18:1/22:6 (closed circles), 18:1/22:5 (open triangles), 18:1/20:4 (dashes), 22:6/22:6 (striped squares), 20:4/22:6 (plus), 22:5/22:6 (open diamonds) are shown in A (control) and B (SCD1 KD). PG 32:0 (closed diamonds), 32:1 (open squares), 34:2 (crosses), 36:1 (closed squares), 36:2 (closed circles), 36:3 (open circles), 34:1 (open triangles), 34:1 (closed triangles) are shown in C (control) and D (SCD1 KD). Mean results are expressed (n = 3) in nmol or pmol of protein loaded onto the sucrose gradient prior to fractionation.
(TIF)

S4 Fig Ratio of monounsaturated acyl chains to structurally similar monounsaturated acyl chains of BMP and PG in the membrane microdomains of control and SCD1KD adipocytes. The ratios of BMP 16:0/16:0 to 16:0/16:1 and PG 32:0 to 32:1 are shown in the DRM (A) and soluble membrane domains (B). Results for control (open bars) and SCD1KD (filled bars) adipocytes are expressed as mean and standard deviation (n = 3). **Significant at p<0.01 (Student’s t-test).
(TIF)
