# Supplementary figures and images for: Stearoyl-CoA Desaturase 1 Is a Key Determinant of Membrane Lipid Composition in 3T3-L1 Adipocytes
Source: PLoS One. 2016 Sep 15;11(9):e0162047. doi: 10.1371/journal.pone.0162047 (PMC5025088; doi:10.1371/journal.pone.0162047)

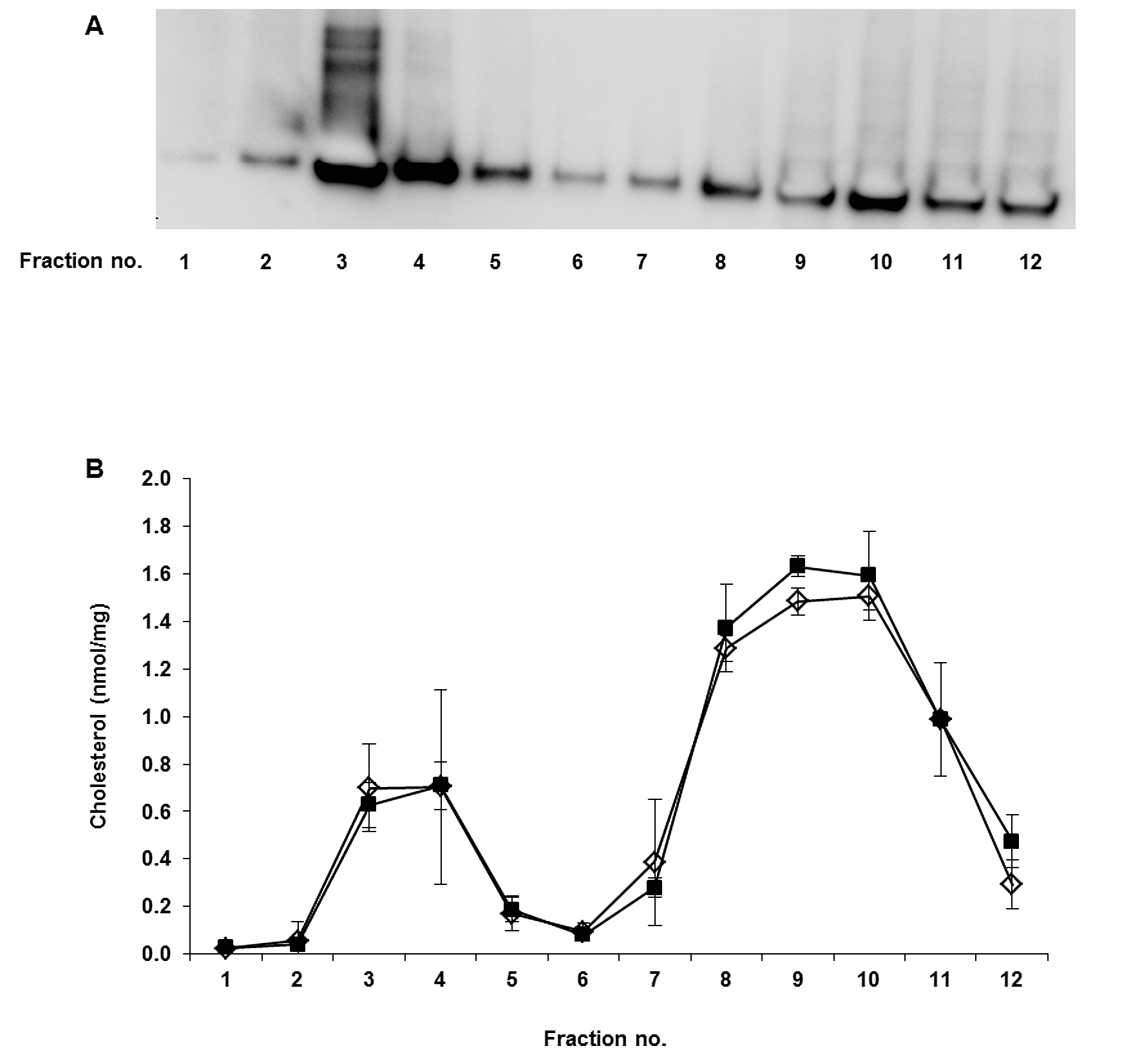

Supplement: S1 Fig — Western blot showing flotillin 1 for each of the membrane fractions isolated from control adipocytes (A). Cholesterol is shown for control (open diamonds) and SCD1 KD (filled squares) adipocytes in B. Results are expressed as mean and standard deviation (n = 3) in nmol of cholesterol per mg of protein loaded onto the gradient prior to fractionation. (TIF) [file pone.0162047.s001.tif]

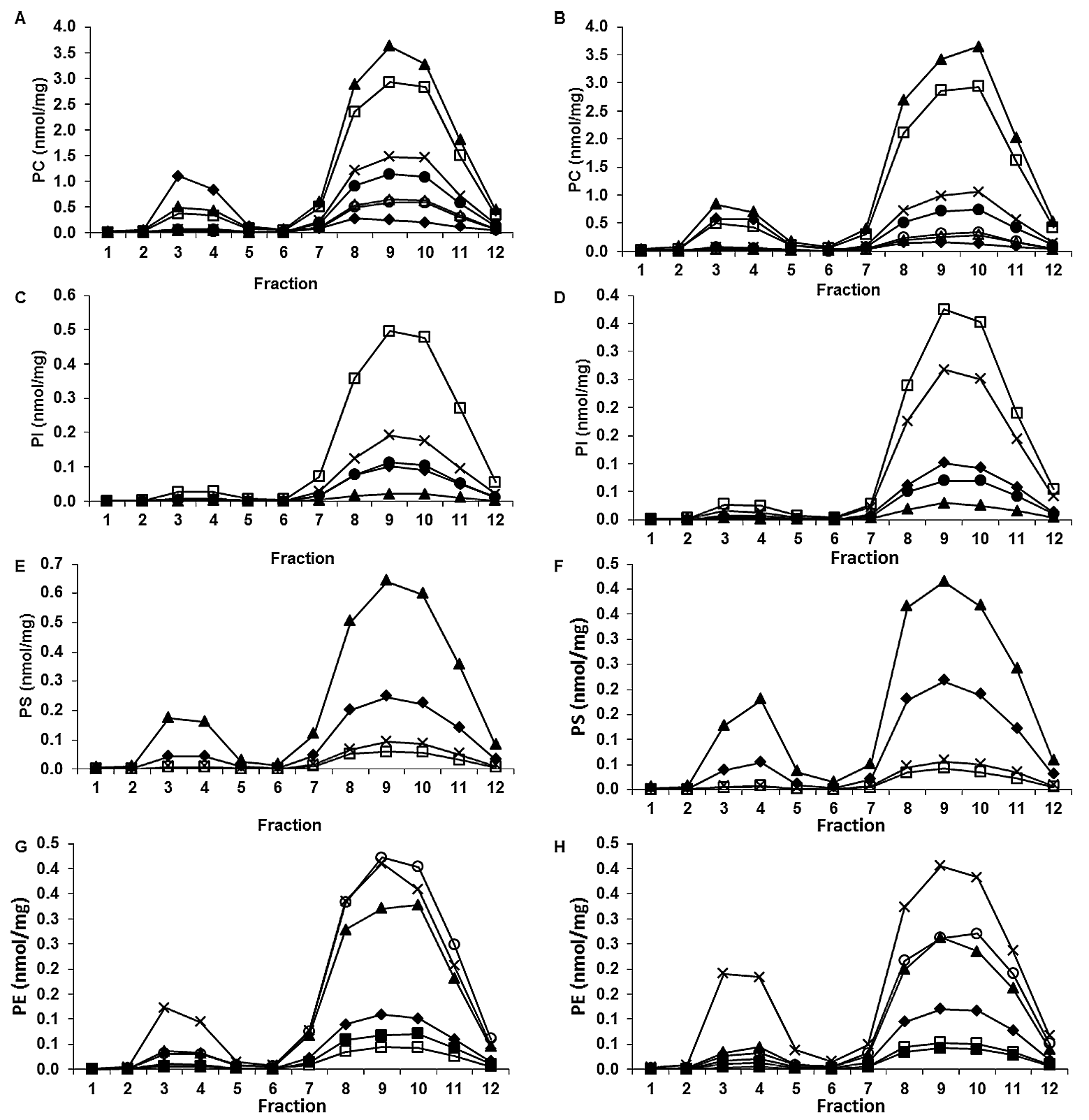

Supplement: S2 Fig — Membrane microdomains were isolated from control 3T3-L1 and SCD1 KD adipocytes and individual species of phospholipids are depicted across the 12 fractions. PC 32:0 (closed diamonds), 32:1 (open squares), 34:1 (closed triangles), 34:2 (crosses), 36:2 (closed circles), 36:4 (open circles) and 38:4 (open triangles) are shown in A (control) and B (SCD1 KD). PI 16:0/20:4 (closed diamonds), 08:0/20:4 (open squares), 18:0/22:45 (closed triangles), 18:1/18:1 (crosses), 18:1/20:4 (closed circles) are shown in C (control) and D (SCD1 KD). PS 18:0/18:2 (closed diamonds), 18:0/20:4 (open squares), 18:1/18:0 (closed triangles), 18:1/18:1) are shown in E (control) and F (SCD1 KD). PE 16:0/22:4 (closed diamonds), 18:0/18:2 (open squares), 18:0/20:4 (closed triangles), 18:1/18:0 (crosses), 18:1/18:1 (open circles), 18:1/20:4 (closed squares) are shown in G (control) and H (SCD1 KD). Mean results are expressed (n = 3) in nmol or pmol of protein loaded onto the sucrose gradient prior to fractionation. (TIF) [file pone.0162047.s002.tif]

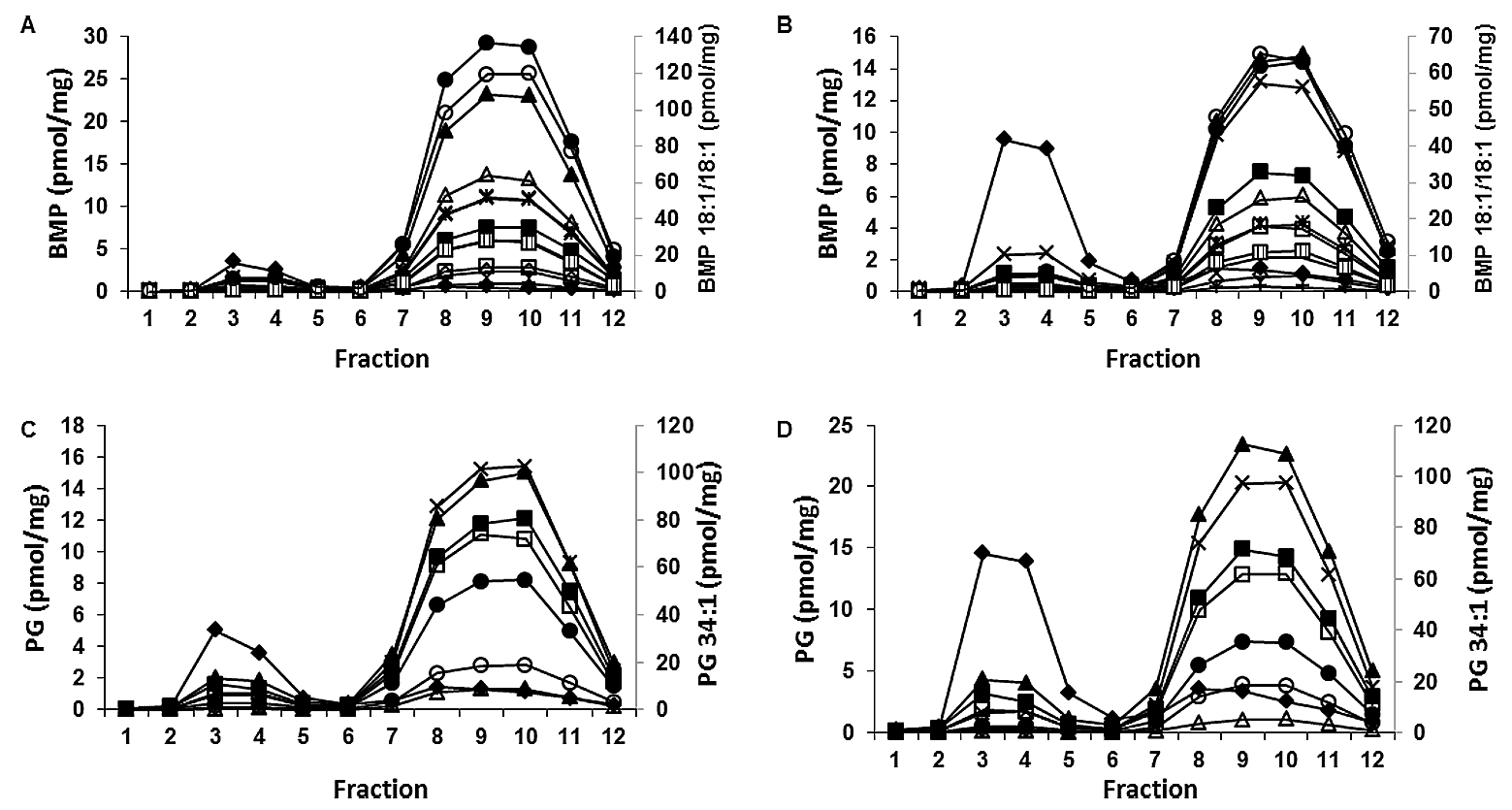

Supplement: S3 Fig — Membrane microdomains were isolated from control 3T3-L1 and SCD1 KD adipocytes and individual species of phospholipids are depicted across the 12 fractions. BMP 16:0/16:0 (closed diamonds), 16:1/16:0 (open squares), 16:1/18:1 (closed triangles), 18:1/16:0 (crosses), 18:1/18:0 (closed squares), 18:1/18:2 (asterisks), 18:1/18:1 (open circles), 18:1/22:6 (closed circles), 18:1/22:5 (open triangles), 18:1/20:4 (dashes), 22:6/22:6 (striped squares), 20:4/22:6 (plus), 22:5/22:6 (open diamonds) are shown in A (control) and B (SCD1 KD). PG 32:0 (closed diamonds), 32:1 (open squares), 34:2 (crosses), 36:1 (closed squares), 36:2 (closed circles), 36:3 (open circles), 34:1 (open triangles), 34:1 (closed triangles) are shown in C (control) and D (SCD1 KD). Mean results are expressed (n = 3) in nmol or pmol of protein loaded onto the sucrose gradient prior to fractionation. (TIF) [file pone.0162047.s003.tif]

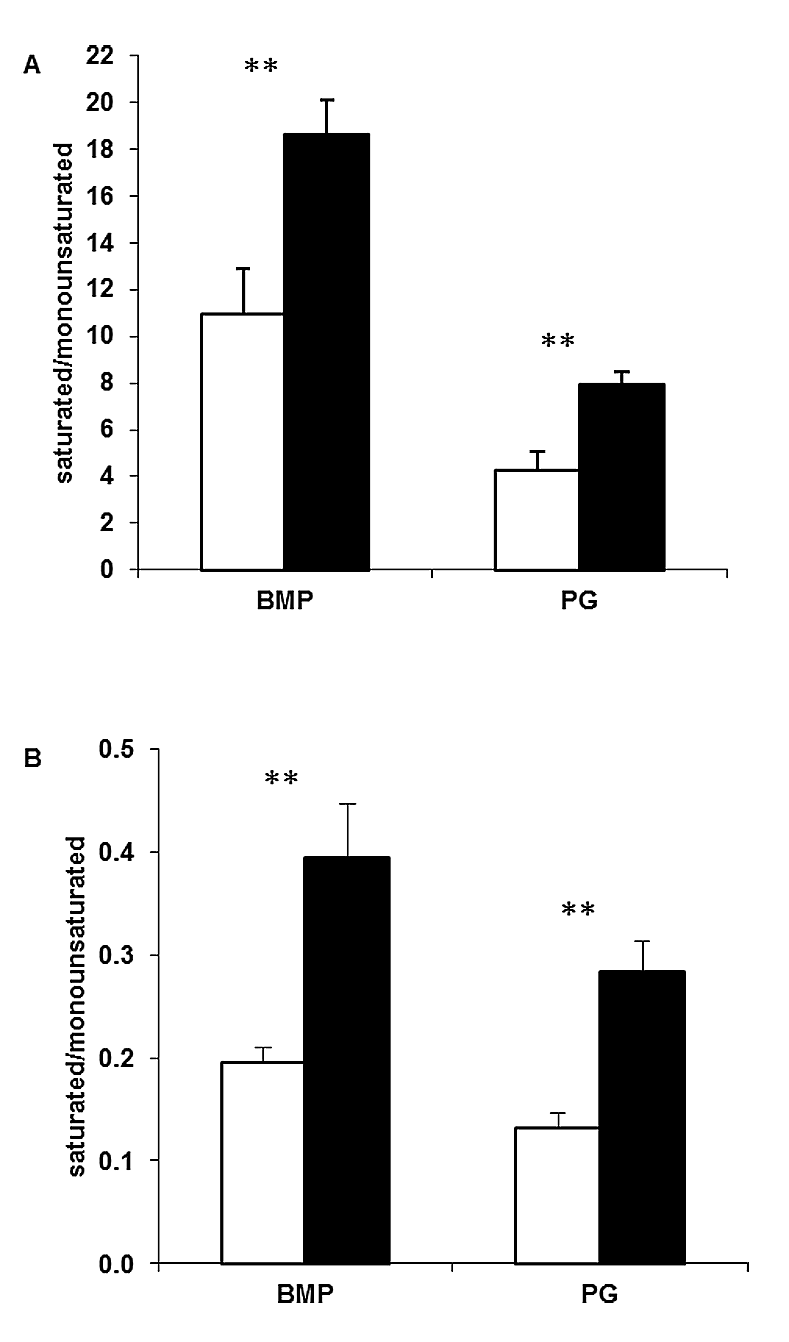

Supplement: S4 Fig — The ratios of BMP 16:0/16:0 to 16:0/16:1 and PG 32:0 to 32:1 are shown in the DRM (A) and soluble membrane domains (B). Results for control (open bars) and SCD1KD (filled bars) adipocytes are expressed as mean and standard deviation (n = 3). **Significant at p<0.01 (Student’s t-test). (TIF) [file pone.0162047.s004.tif]
